# Comparison of Four *Streptococcus pneumoniae* Urinary Antigen Tests Using Automated Readers

**DOI:** 10.3390/microorganisms9040827

**Published:** 2021-04-13

**Authors:** Alicia Yoke Wei Wong, Alexander Tony Arvind Johnsson, Karolina Ininbergs, Simon Athlin, Volkan Özenci

**Affiliations:** 1 Department of Laboratory Medicine, Division of Clinical Microbiology, Karolinska Institutet, 141 86 Stockholm, Sweden; karolina.ininbergs@sll.se; 2 Department of Clinical Microbiology, Karolinska University Hospital, Huddinge, 141 86 Stockholm, Sweden; alexander.johnsson@sll.se; 3 Department of Clinical Microbiology, Karolinska University Hospital, Solna, 171 76 Stockholm, Sweden; 4 Faculty of Medicine and Health, School of Medical Sciences, Örebro University, 701 82 Örebro, Sweden; simon.athlin@oru.se

**Keywords:** *Streptococcus pneumoniae*, pneumococcal antigen, pneumonia, urinary antigen test (UAT), BinaxNOW, ImmuView, Sofia, STANDARD F

## Abstract

*Streptococcus pneumoniae* urinary antigen tests (UATs) may be interpreted using automatic readers to potentially automate sample incubation and provide standardized results reading. Here, we evaluated four UATs the BinaxNOW *S. pneumoniae* Antigen Card (Abbott, Chicago, IL, USA), ImmuView *S. pneumoniae* and Legionella (SSI Diagnostica, Hillerød, Denmark), STANDARD F *S. pneumoniae* Ag FIA (SD Biosensor, Gyeonggi, South Korea), and Sofia *S. pneumoniae* FIA (Quidel Corporation, San Diego, CA, USA) with their respective benchtop readers for their ability to detect *S. pneumoniae* urinary antigen. We found that these assays had a sensitivity of 76.9–86.5%, and specificity of 84.2–89.7%, with no significant difference found among the four UATs. The assays had a high level of agreement with each other, with 84.5% of samples testing consistently across all four assays. The automatically and visually read test results from the two immunochromatographic assays, BinaxNOW and ImmuView, were compared and showed excellent agreement between the two types of reading. Immunofluorescent-based assays, Sofia and STANDARD F, had significantly less time to detect compared to the two immunochromatographic assays due to having less assay setup procedures and shorter sample incubation times. In conclusion, the four UATs performed similarly in the detection of *S. pneumoniae* urinary antigen, and readers can bring increased flexibility to running UATs in the clinical routine.

## 1. Introduction

*Streptococcus pneumoniae* is a leading cause of bacterial infections such as pneumonia, otitis media, sepsis, and meningitis [1], responsible for invasive infections in 10 to 100 cases per 100,000 people in Europe and United States per year [2]. Traditional culture-based diagnostic methods for blood, cerebrospinal fluid (CSF), or respiratory tract samples remain standard methods for diagnosis, but the procedures have a lengthy time-to-detection (TTD) and are labor-intensive. Since early detection of *S. pneumoniae* is crucial in patients with invasive disease, there is a need for reliable, rapid, and user-friendly diagnostic assays.

Previous studies of immunochromatography-based *S. pneumoniae* urine antigen tests (UATs) have reported sensitivities of 62–73% and specificities of 86–100% [3,4,5,6,7]. Immunofluorescent-based UATs have shown higher sensitivity compared to immunochromatography-based UATs [5,6]. While automated readers are required for the interpretation of immunofluorescence-based UATs, they are not necessary for interpretation of immunochromatography-based *S. pneumoniae* UATs but may be used. We have recently showed a high agreement between automatically and visually read BinaxNOW *S. pneumoniae* UAT [8], and another study has demonstrated similar results also for the ImmuView *S. pneumoniae* and *L. pneumophila* UAT [9]. The use of readers could potentially automate sample incubation and results interpretation.

Here, we compare the automatic interpretation of two immunochromatographic UATs, the BinaxNOW *S. pneumoniae* Antigen Card (BinaxNOW) and ImmuView *S. pneumoniae* and *L. pneumophila* (ImmuView), and two fluorescent based UATs, Sofia *S. pneumoniae* Fluorescent Immunoassay (FIA) (Sofia) and STANDARD F *S. pneumoniae* Ag FIA (STANDARD F). The present study compares the performance of four UATs in conjunction with their respective readers. Samples were tested simultaneously with the four UATs. For the immunochromatography-based UATs, the results obtained from the readers were compared to visually interpreted results. In addition, we examined the performance of antigen detection after refreezing, and compared time to result and user friendliness between the UATs.

## 2. Materials and Methods

### 2.1. Samples

Urine samples from adult patients ≥18 years old were obtained from the clinical routine at the Department of Clinical Microbiology at Karolinska University Hospital Huddinge (Stockholm, Sweden) and the Department of Laboratory Medicine at Örebro University Hospital (Örebro, Sweden). Inclusion criteria were urine samples from bacteremic patients with simultaneously collected blood culture and/or respiratory tract culture ±2 days of urine sample collection. Blood and respiratory culture results were obtained from a Laboratory Information System. Blood and respiratory tract samples were cultured according to standard clinical protocols. Briefly, after Gram stain was performed, positive blood culture bottles with diplococci were subsequently sub-cultured on blood, chocolate, and crystal violet blood (in-house production, Karolinska University Hospital Huddinge, Stockholm, Sweden) agar plates. Respiratory samples were cultured on relevant agar plates including crystal violet blood agar plates. Identification of *S. pneumoniae* was performed according to optocin susceptibility. Urine samples from patients with blood and/or respiratory sample cultures positive for *S. pneumoniae* were used as positive cases Urine samples from patients negative for *S. pneumoniae* in blood and respiratory tract cultures were classified as negative controls. All samples were anonymized after culture results had been retrieved. Samples were stored at −80 °C and were thawed in room temperature prior to testing. Each patient sample was tested with all four UATs (BinaxNOW, ImmuView, Sofia, STANDARD F) and immediately re-frozen. In order to analyze the effect of long-term storage after re-freezing, the urine samples were frozen at −80 °C for another 4 months before re-testing with the four antigen tests.

### 2.2. Urinary Antigen Tests (UATs)

UATs from the four manufacturers were used together with their respective readers (Table 1). For the BinaxNOW, Sofia, and STANDARD F, samples were incubated and read by the readers on “Walk Away” mode. UAT setup procedures are briefly described as follows. BinaxNOW setup included dipping the included swab into the sample, inserting it into the sample card, adding reagent buffer, and then inserting the sample card into the reader for incubation and interpretation as per manufacturer’s instructions. For Sofia and STANDARD F, the sample was introduced to the sample well on the cassette by a dropper, and inserted into their respective reader for incubation and interpretation as per manufacturers’ instructions. For ImmuView, three drops of sample were added to the included polypropylene tube followed by addition of two drops of reagent buffer and gently mixed. The UAT strip was then inserted into the tube, and incubation was timed manually before the UAT was inserted into the reader for interpretation. Sample incubation time was 15 min for BinaxNOW and ImmuView, and 10 min for Sofia and STANDARD F. Samples were run simultaneously with each of the four UATs, and the results provided by the readers were noted. The readers indicated if the test was valid, and if the sample result was positive or negative for *S. pneumoniae*. If a result obtained for a sample was invalid or the reader displayed an error message, the sample was re-analyzed using a new UAT. The STANDARD F200 Analyzer also indicated the cut-off index (COI) value along with the sample result. In addition, the test results were visually interpreted by two independent researchers for BinaxNOW and ImmuView after automated reading was performed. The appearance of a correctly colored band that was visible to the naked eye after 15 min was considered as a positive result. If the visual interpretations of the UAT differed between researchers, the result that was consistent with automated reading was used. The time to result was registered by measuring the time from the start of “Walk Away” mode (except for ImmuView) to when the sample result was presented (printed out or displayed) by the reader.

### 2.3. Statistical Analysis

Sensitivities and specificities of the UATs were determined, and the confidence intervals (CI) were computed using the Wilson–Brown method. The significance of differences between tests were computed using Cochran’s Q test (*χ*^2^ (3)) using Microsoft Excel version 16.40 [10]. Differences in time to result between UATs were analyzed by calculating mean time from three measurements for each UAT and performing one-way ANOVA followed by Bonferroni’s multiple comparisons test. Statistical calculations were performed using Prism 8 (GraphPad Software LLC, San Diego, CA, USA). A *p*-value of ≤0.05 was considered as statistically significant. A CI of 95% was used for statistical precision.

## 3. Results

### 3.1. Sample Characteristics

**Figure 1:**
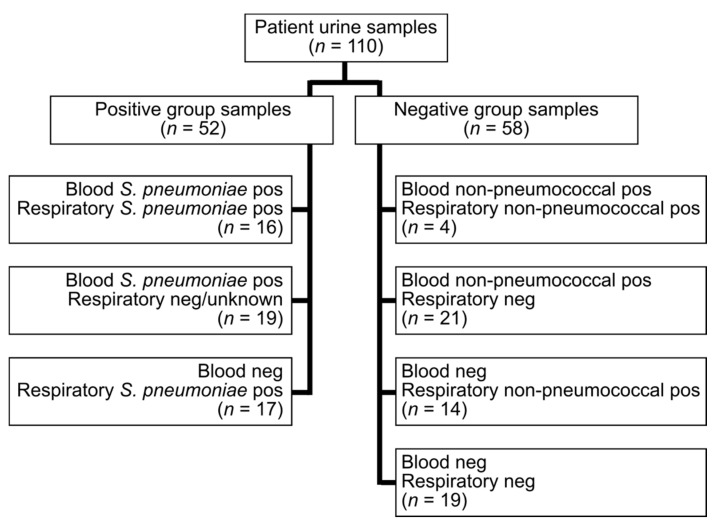
Blood and respiratory tract culture results for patients included in the study. Pos, positive; neg, negative.

A total of 110 samples were analyzed, 52 were positive cases and 58 negative controls (Figure 1, Tables S1 and S2).

### 3.2. Sensitivity of S. pneumoniae UATs

The results from testing *S. pneumoniae* positive urine samples with the four UATs are presented in Table 2 and Table S1. Sensitivities for BinaxNOW, ImmuView, Sofia, and STANDARD F were 82.7% (95% CI, 70.2–90.6%), 76.9% (95% CI, 63.9–86.3%), 86.5% (95% CI, 74.7–93.3%), and 80.8% (95 % CI, 68.1–89.2%), respectively. Cochran’s Q test did not indicate any significant differences between sensitivity rates of the four assays (*χ*^2^ (3) Q = 5.57, *p* = 0.13) (Table 2). A total of 14 positive samples showed false negative or discordant results by one or more UATs (Table 3).

### 3.3. Specificity of S. pneumoniae UATs

The results from testing negative control urine samples with the four UATs are presented in Table 4 and Table S2. Specificities for BinaxNOW and Sofia were both 89.7% (95% CI, 79.2–95.2%), and for ImmuView and STANDARD F 84.5% (95% CI, 73.1–91.6%) and 84.2% (95% CI, 72.6–91.5%), respectively. Cochran’s Q test did not indicate any significant differences between the specificities of the four assays (*χ*^2^ (3) Q = 4.50, *p* = 0.21) (Table 4). A total of 13 negative samples showed false positive or discordant results by one or more UATs, including one sample that tested invalid by Sofia (N56) (Table 5).

### 3.4. Agreement of Results between UATs

**Figure 2:**
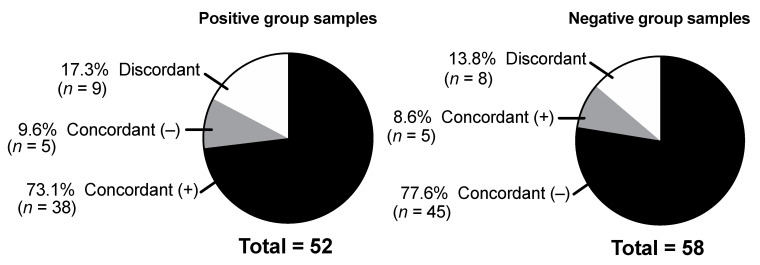
Agreement of results between the four *Streptococcus pneumoniae* urine antigen tests.

Excellent agreement between the four UATs was observed. In positive group samples, there was 82.7% concordance, where 73.1% of samples tested positive and 9.6% tested negative in all four UATs. For the negative group samples, the concordance rate was 86.2%. In total, 77.6% of samples tested negative, and 8.6% tested positive in all four assays. Overall, the four assays had discordant results in 17/110 (15.5%) samples (Figure 2).

### 3.5. Agreement between Visual and Automatic Reading of UATs

Visual readings of the BinaxNOW UAT by the two researchers were 100% in agreement with each other, while that for the ImmuView UAT was mostly in agreement apart from one sample (P19 from Table 3 and Table S1), where one researcher interpreted the pneumococci result as negative while the other interpreted it as positive. There was a very good agreement between visual and automatic reading for BinaxNOW. Only two samples (P35 from Table S1, and N17 from Table S2) that were interpreted as negative visually by both researchers was positive by the DIGIVAL reader. Similarly, very good agreement between visual and automatic reading of ImmuView was also observed. Only one sample (P5 from Table S1) that was interpreted as positive visually by both researchers but negative by the ImmuView reader.

### 3.6. Assay Performance after Long-Time Storage

**Figure 3:**
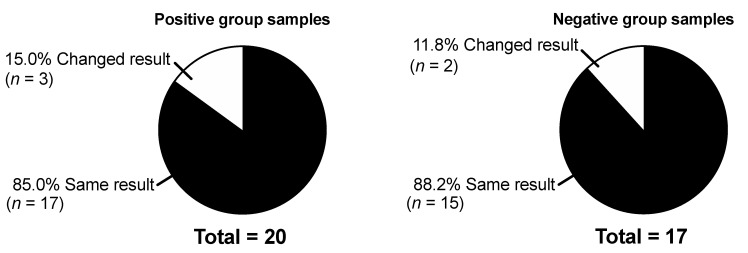
Proportion of samples with the same result, or a changed result, in one or more of the four *Streptococcus pneumoniae* urine antigen tests after four months of storage at −80 °C.

For performance analysis after long-time freezing, 37 samples (positive cases, *n* = 20; negative controls, *n* = 17) were randomly selected for re-testing with BinaxNOW, ImmuView, and STANDARD F after four months of storage at −80 °C. For 32 (86.5%) samples, the UATs showed the same result after storage, while for five (13.5%) samples, the results changed (Figure 3 and Table S3).

### 3.7. Time to Result and User Friendliness

**Figure 4:**
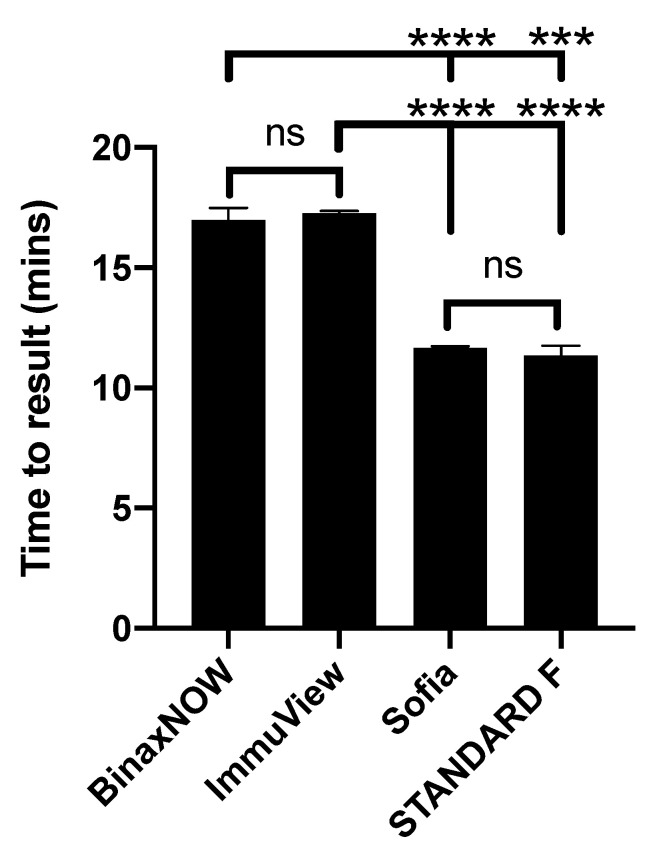
Time to result for four *Streptococcus pneumoniae* urine antigen tests. (*** *p* ≤ 0.001; **** *p* ≤ 0.0001).

The mean time to result for BinaxNOW, ImmuView, Sofia, and STANDARD F were 17.0, 17.3, 11.7, and 11.3 min, respectively, with significant differences between immunochromatographic-based and immunofluorescent-based assays that could attributed to more steps required to set up a test, thus, more hands-on time (Figure 4).

Observations with regard to the UAT usage, barcode scanning, and result presentation were noted for assessing user friendliness. While the UATs of the BinaxNOW, Sofia, and STANDARD F were enclosed, thus, reducing the chance of samples coming directly into contact with the readers, the ImmuView UAT was exposed, which led to frequent cleaning of the drawer that came into contact with the sample. The readers for BinaxNOW, Sofia, and STANDARD F were equipped with barcode scanners, either fixed on either a stand or at the side of the reader, which facilitated hands-free scanning (BinaxNOW and Sofia), or as a handheld barcode scanner with a button for activating scanning (STANDARD F). Lastly, the readers for BinaxNOW, Sofia, and STANDARD F had the option to print results, while results from the ImmuView reader is read on the display only. The BinaxNOW reader can be connected to a small external printer, while Sofia and STANDARD F readers had built-in printers.

## 4. Discussion

The present study evaluated the performance of four *S. pneumoniae* urinary antigen detection assays using automatic readers for interpretation of test results. The UATs demonstrated similar sensitivities and specificities of 76.9–86.5% and 84.2–89.7%, respectively. The test results showed high concordance across different UATs, as well as a high level of agreement between automatically and visually read test results. Furthermore, our investigations showed that a majority of samples retained the same result after re-freezing and long storage. Lastly, the four UATs were generally easy to use, providing features such as barcode scanning, automated incubation, and results printing. However, immunochromatographic-based assays have more setup procedures compared to immunofluorescent-based assays and, hence, longer hands-on time.

Our findings of *S. pneumoniae* UAT performance are within the range of sensitivities and specificities from previous studies [3,4,5,6,7,11]. Additionally, the agreement between automatically and visually interpreted results for BinaxNOW and ImmuView was excellent. However, there were no difference in sensitivity yields between immunochromatography-based and immunofluorescent-based assays, unlike what was previously reported [5,6]. Accordingly, we have previously reported that the automatically read Sofia showed similar performance rates, as the visually read BinaxNOW and ImmuView on frozen samples from bacteremic patients as well as on consecutive non-frozen urine samples [8]. The reason for discrepant study results may be due to differences in sample collection, storage, and/or testing, which must be sufficiently clarified throughout the study protocol in order to make results comparable across studies.

All four UATs failed to detect *S. pneumoniae* antigen in 5/52 urine samples among the pneumococcal cases, and one or more of the UATs were unable to detect pneumococcal antigen in another nine samples similar to what was found previously [12]. Most likely, low levels of pneumococcal antigen in urine is the reason for negative test results.

In 10.3–15.5% of negative control samples, false positive results were obtained, mainly from patients with either Gram-negative infections including patients with blood cultures positive for *B. fragilis* (*n* = 1), *E. coli* (*n* = 2), and *P. mirabilis* (*n* = 1) or respiratory tract cultures positive for *H. influenzae* (*n* = 2), *K. pneumoniae* (*n* = 1), and *M. catarrhalis* (n = 1). In only two cases, false positive results were obtained from patients with negative blood and respiratory tract cultures. Prior studies have also reported false positive results on samples from patients infected by Gram-negative bacteria or alpha-streptococci [13,14,15]. Preheating of the urine may help to increase the specificity of urinary antigen assays [6,15] but was not performed for this study as the majority of manufacturers did not recommend it.

An important factor for assay reliability is the rate of invalid test results. In the present study, only 1/440 (0.2%) of all UATs performed showed an invalid result, as it was invalid for one Sofia UAT. We previously reported a higher number of invalid test results for *S. pneumoniae* urine antigen assays, in particular for the ImmuView [3] and Sofia UATs [8], which could be due to presence of interfering substances in samples or high sample viscosities.

To our knowledge, this is the first time the four *S. pneumoniae* UATs were evaluated simultaneously with their respective readers, including the first evaluation of STANDARD F performance for *S. pneumoniae* antigen detection. Only the STANDARD F reader presented COI values as a way to quantify the concentration of pneumococcal antigen in urine samples in addition to the dichotomous test results (positive or negative). The importance of COI values in clinical routine has not been studied as we know; however, as the other readers did not provide quantifiable results, we were unable to compare result signal intensity across the four assays. Automated readers could bring convenience to the workflow, as they provide onscreen prompts on assay setup procedures. The BinaxNOW, Sofia, and STANDARD F readers also provide the alternatives to run in either “Walk Away” or “Read Now” mode. This allows the flexibility to choose whether to automatically incubate and read single samples, or to manually incubate multiple samples and read them in quick succession with the reader. Therefore, although automatic reading did not enhance the sensitivity of the assays in the present material, readers may facilitate flexibility and time-efficiency in the workflow.

We previously reported various aspects of user friendliness of the BinaxNOW, ImmuView, Sofia, and STANDARD F *Legionella* UATs that are also applicable to this study [16]. Regarding the time to result of the assays, the immunochromatographic-based assays had longer time to result as compared to the immunofluorescent-based assays due to more assay setup procedures as well as longer incubation time. The longer hands-on time and time to result for immunochromatographic-based assays could perhaps be viewed as a trade-off to increased flexibility, since these assays do not require readers to obtain results. The option to interpret assay results visually could be more ideal for low-resource clinical settings.

A limitation of our study is that we used frozen urine samples for evaluation of test performances. The pneumococcal cell wall polysaccharide antigen is considered to be temperature stable [8,17,18]. A previous study evaluating the performance of BinaxNOW UAT showed similar test results on both fresh and frozen unconcentrated urine samples where the frozen samples were only thawed once [19]. We do not expect that storage at −80 °C would have a significant impact on sample results if the sample was thawed once; however, our results with re-frozen samples show that subsequent freeze–thaw cycles have the potential to change results in a small subset of samples.

Another limitation is that no additional patient information other than blood and respiratory tract culture results were obtained from the Laboratory Information System. This made it difficult interpreting false positive and false negative results obtained, and we were not able to conclude how long *S. pneumoniae* urinary antigen detection lasts after an infection.

In conclusion, the present study compares for the first time four *S. pneumoniae* UATs with automatic interpretation using their respective readers on defined clinical samples. The four assays performed similarly and are largely in agreement for the detection of *S. pneumoniae* urinary antigen. The use of a reader, availability of barcode scanning, and automated sample incubation brings increased flexibility, ease of use, and standardization in performing UATs in the clinical routine, thus improving patient diagnosis through increased efficiency and decreased probability of errors.

## Figures and Tables

**Table 1:** *Streptococcus pneumoniae* urine antigen assays evaluated in the study.

Assay	Commercial Name	Reader	Manufacturer
BinaxNOW	BinaxNOW *S. pneumoniae* Antigen Card	DIGIVAL instrument	Abbott (Chicago, IL, USA)
ImmuView	ImmuView *S. pneumoniae* and *Legionella*	ImmuView Reader	SSI Diagnostica (Hillerød, Denmark)
Sofia	Sofia *S. pneumoniae* FIA	Sofia FIA Analyzer	Quidel Corporation (San Diego, CA, USA)
STANDARD F	STANDARD F *S. pneumoniae* Ag FIA	STANDARD F200 Analyzer	SD Biosensor (Gyeonggi, South Korea)

FIA, fluorescence immunoassay.

**Table 2:** Sensitivity of four *Streptococcus pneumoniae* urine antigen tests in patients with pneumococcal infection.

Assay	UAT Positive	UAT Negative	Sensitivity (95% CI)
BinaxNOW	43	9	82.7 (70.2–90.6)
ImmuView	40	12	76.9 (63.9–86.3)
Sofia	45	7	86.5 (74.7–93.3)
STANDARD F	42	10	80.8 (68.1–89.2)
*χ*^2^ (3)			Q = 5.57 (*p* = 0.13)

Urine samples from patients with *S. pneumoniae* positive blood and/or respiratory tract cultures.

**Table 3:** False negative and discordant test results by four *Streptococcus pneumoniae* urine antigen tests in 14 patients with pneumococcal infection.

Sample	Blood Culture Result	Respiratory Culture Result	BinaxNOW	ImmuView	Sofia	STANDARD F
P15	*Streptococcus pneumoniae*	*Streptococcus pneumoniae*	Negative	Negative	Negative	Negative
P28	*Streptococcus pneumoniae*	Negative (normal flora)	Negative	Negative	Negative	Negative
P23	*Streptococcus pneumoniae*	Negative (normal flora)	Negative	Negative	Negative	Negative
P51	Negative	*Streptococcus pneumoniae*	Negative	Negative	Negative	Negative
P36	*Escherichia coli*	*Streptococcus pneumoniae*	Negative	Negative	Negative	Negative
P3	*Streptococcus pneumoniae*	*Streptococcus pneumoniae*, *Staphylococcus aureus*	Negative	Positive	Negative	Negative
P24	*Streptococcus pneumoniae*	Negative (normal flora)	Negative	Negative	Positive	Negative
P27	*Streptococcus pneumoniae*	Negative (normal flora)	Negative	Negative	Positive	Negative
P35	*Streptococcus pneumoniae*	Not determined	Positive	Negative	Negative	Negative
P38	Negative	*Streptococcus pneumoniae*	Negative	Positive	Positive	Negative
P5	*Streptococcus pneumoniae*	*Streptococcus pneumoniae*	Positive	Negative	Positive	Positive
P19	*Streptococcus pneumoniae*	*Staphylococcus aureus*	Positive	Negative	Positive	Positive
P34	*Streptococcus pneumoniae*	Not determined	Positive	Negative	Positive	Positive
P42	Negative	*Streptococcus pneumoniae*	Positive	Negative	Positive	Positive

**Table 4:** Specificity of four *Streptococcus pneumoniae* urine antigen tests in patients without pneumococcal infection.

Assay	UAT Negative	UAT Positive	Invalid/Error	Sensitivity (95% CI)
BinaxNOW	52	6	0	89.7 (79.2–95.2)
ImmuView	49	9	0	84.5 (73.1–91.6)
Sofia	48	9	1	84.2 (72.6–91.5)
STANDARD F	52	6	0	89.7 (79.2–95.2)
*χ*^2^ (3)				Q = 4.50 (*p* = 0.70)

**Table 5:** False positive and discordant test results by four *Streptococcus pneumoniae* urine antigen tests in 13 patients without pneumococcal infection.

Sample	Blood Culture Result	Respiratory Culture Result	BinaxNOW	ImmuView	Sofia	STANDARD F
N5	*Bacteroides fragilis*	Negative (normal flora)	Positive	Positive	Positive	Positive
N32	Negative	*Klebsiella pneumoniae*	Positive	Positive	Positive	Positive
N38	Negative	*Moraxella catarrhalis*	Positive	Positive	Positive	Positive
N31	Negative	*Hemophilus influenzae*	Positive	Positive	Positive	Positive
N47	Negative	Negative (normal flora)	Positive	Positive	Positive	Positive
N27	Negative	*Hemophilus influenzae*	Negative	Positive	Positive	Negative
N43	Negative	Negative (normal flora)	Negative	Positive	Positive	Negative
N56	Negative	Negative (normal flora)	Negative	Negative	Invalid	Positive
N23	*Staphylococcus epidermidis*	Negative (normal flora)	Negative	Positive	Negative	Negative
N11	*Escherichia coli*	Negative (normal flora)	Negative	Negative	Positive	Negative
N17	*Proteus mirabilis*	Negative (normal flora)	Positive	Negative	Negative	Negative
N6	*Escherichia coli*	Negative (normal flora)	Negative	Negative	Positive	Negative
N33	Negative	*Moraxella catarrhalis*	Negative	Positive	Negative	Negative

## Data Availability

The data presented in this study are openly available upon request.

## Supplementary Materials

The following are available online at https://www.mdpi.com/article/10.3390/microorganisms9040827/s1, Table S1: Streptococcus pneumoniae UAT results for positive group patient urine samples, Table S2: Streptococcus pneumoniae UAT results for negative group patient urine samples., Table S3: Results of *Streptococcus pneumoniae* UATs after re-freezing and four months of storage.
