# Supplementary material for: Comparison of Four Streptococcus pneumoniae Urinary Antigen Tests Using Automated Readers
Source: Microorganisms. 2021 Apr 13;9(4):827. doi: 10.3390/microorganisms9040827 (PMC8070120; doi:10.3390/microorganisms9040827)
Supplement: Supplementary file 1 [file microorganisms-09-00827-s001.pdf]

microorganisms

MDPI

Supplementary Data

Comparison of four Streptococcus pneumonia urinary antigen tests interpreted using automated readers

Alicia Y. W. Wong, Alexander T. A. Johnsson, Karolina Iningbergs, Aina Iversen, Simon Athlin, and Volkan Özenci

Table S1. *Streptococcus pneumoniae UAT results for positive group patient urine samples*

Sample	Blood culture result	Respiratory tract culture result	Respiratory sample type	UAT result			
				BinaxNOW	ImmuView	STANDARD F	Sofia
P1	*Streptococcus pneumoniae*	*Streptococcus pneumoniae*, *Haemophilus influenzae*	Sputum	Positive	Positive	Positive	Positive
P2	*Streptococcus pneumoniae*	*Streptococcus pneumoniae*, *Moraxella cattarhalis*	Sputum	Positive	Positive	Positive	Positive
P3	*Streptococcus pneumoniae*	*Streptococcus pneumoniae*, *Staphylococcus aureus*	Nasopharynx, sputum	Negative	Positive	Negative	Negative
P4	*Streptococcus pneumoniae*	*Streptococcus pneumoniae*	Nasopharynx, sputum	Positive	Positive	Positive	Positive
P5	*Streptococcus pneumoniae*	*Streptococcus pneumoniae*	Nasopharynx	Positive	Negative	Positive	Positive
P6	*Streptococcus pneumoniae*	*Streptococcus pneumoniae*	Nasopharynx	Positive	Positive	Positive	Positive
P7	*Streptococcus pneumoniae*	*Streptococcus pneumoniae*	Sputum	Positive	Positive	Positive	Positive
P8	*Streptococcus pneumoniae*	*Streptococcus pneumoniae*	Sputum	Positive	Positive	Positive	Positive
P9	*Streptococcus pneumoniae*	*Streptococcus pneumoniae*	Nasopharynx	Positive	Positive	Positive	Positive
P10	*Streptococcus pneumoniae*	*Streptococcus pneumoniae*	Nasopharynx, sputum	Positive	Positive	Positive	Positive
P11	*Streptococcus pneumoniae*	*Streptococcus pneumoniae*	Nasopharynx	Positive	Positive	Positive	Positive
P12	*Streptococcus pneumoniae*	*Streptococcus pneumoniae*	Nasopharynx	Positive	Positive	Positive	Positive
P13	*Streptococcus pneumoniae*	*Streptococcus pneumoniae*	Nasopharynx, sputum	Positive	Positive	Positive	Positive
P14	*Streptococcus pneumoniae*	*Streptococcus pneumoniae*	Nasopharynx	Positive	Positive	Positive	Positive
P15	*Streptococcus pneumoniae*	*Streptococcus pneumoniae*	Nasopharynx	Negative	Negative	Negative	Negative
P16	*Streptococcus pneumoniae*	*Streptococcus pneumoniae*	Nasopharynx	Positive	Positive	Positive	Positive
P17	*Streptococcus pneumoniae*	*Moraxella cattarhalis*	Nasopharynx	Positive	Positive	Positive	Positive
P18	*Streptococcus pneumoniae*	*Proteus* spp.	Nasopharynx	Positive	Positive	Positive	Positive
P19	*Streptococcus pneumoniae*	*Staphylococcus aureus*	Nasopharynx	Positive	Negative	Positive	Positive
P20	*Streptococcus pneumoniae*	*Staphylococcus aureus*	Sputum	Positive	Positive	Positive	Positive
P21	*Streptococcus pneumoniae*	Negative (normal flora)	Sputum	Positive	Positive	Positive	Positive
P22	*Streptococcus pneumoniae*	Negative (normal flora)	Nasopharynx	Positive	Positive	Positive	Positive
P23	*Streptococcus pneumoniae*	Negative (normal flora)	Nasopharynx	Negative	Negative	Negative	Negative
P24	*Streptococcus pneumoniae*	Not determined	-	Negative	Negative	Negative	Positive

Supplementary data – Wong et al. 2021.
Comparison of four Streptococcus pneumonia urinary antigen tests interpreted using automated readers

Sample	Blood culture result	Respiratory tract culture result	Respiratory sample type	UAT result			
				BinaxNOW	ImmuView	STANDARD F	Sofia
P25	*Streptococcus pneumoniae*	Not determined	-	Positive	Positive	Positive	Positive
P26	*Streptococcus pneumoniae*	Not determined	-	Positive	Positive	Positive	Positive
P27	*Streptococcus pneumoniae*	Not determined	-	Negative	Negative	Negative	Positive
P28	*Streptococcus pneumoniae*	Not determined	-	Negative	Negative	Negative	Negative
P29	*Streptococcus pneumoniae*	Not determined	-	Positive	Positive	Positive	Positive
P30	*Streptococcus pneumoniae*	Not determined	-	Positive	Positive	Positive	Positive
P31	*Streptococcus pneumoniae*	Not determined	-	Positive	Positive	Positive	Positive
P32	*Streptococcus pneumoniae*	Not determined	-	Positive	Positive	Positive	Positive
P33	*Streptococcus pneumoniae*	Not determined	-	Positive	Positive	Positive	Positive
P34	*Streptococcus pneumoniae*	Not determined	-	Positive	Negative	Positive	Positive
P35	*Streptococcus pneumoniae*	Not determined	-	Positive	Negative	Negative	Negative
P36	*Escherichia coli*	*Streptococcus pneumoniae*	Nasopharynx	Negative	Negative	Negative	Negative
P37	Negative	*Streptococcus pneumoniae*	Nasopharynx	Positive	Positive	Positive	Positive
P38	Negative	*Streptococcus pneumoniae*	Nasopharynx, sputum	Negative	Positive	Negative	Positive
P39	Negative	*Streptococcus pneumoniae*	Sputum	Positive	Positive	Positive	Positive
P40	Negative	*Streptococcus pneumoniae*	Nasopharynx, sputum	Positive	Positive	Positive	Positive
P41	Negative	*Streptococcus pneumoniae*	Sputum	Positive	Positive	Positive	Positive
P42	Negative	*Streptococcus pneumoniae*	Nasopharynx	Positive	Negative	Positive	Positive
P43	Negative	*Streptococcus pneumoniae*	Nasopharynx	Positive	Positive	Positive	Positive
P44	Negative	*Streptococcus pneumoniae*	Nasopharynx, sputum	Positive	Positive	Positive	Positive
P45	Negative	*Streptococcus pneumoniae*	Nasopharynx	Positive	Positive	Positive	Positive
P46	Negative	*Streptococcus pneumoniae*	Nasopharynx	Positive	Positive	Positive	Positive
P47	Negative	*Streptococcus pneumoniae*	Nasopharynx	Positive	Positive	Positive	Positive
P48	Negative	*Streptococcus pneumoniae*	Sputum	Positive	Positive	Positive	Positive
P49	Negative	*Streptococcus pneumoniae*	Nasopharynx	Positive	Positive	Positive	Positive
P50	Negative	*Streptococcus pneumoniae*	Nasopharynx	Positive	Positive	Positive	Positive
P51	Negative	*Streptococcus pneumoniae*	Sputum	Negative	Negative	Negative	Negative
P52	Negative	*Streptococcus pneumoniae*	Nasopharynx	Positive	Positive	Positive	Positive

Table S2. Streptococcus pneumoniae UAT results for negative group patient urine samples

Sample	Blood culture result	Respiratory tract culture result	UAT result			
			BinaxNOW	ImmuView	STANDARD F	Sofia
N1	Group B Streptococcus	*Staphylococcus aureus*, *Moraxella catarrhalis*	Negative	Negative	Negative	Negative
N2	*Providencia rettgeri*	*Staphylococcus aureus*	Negative	Negative	Negative	Negative
N3	*Pseudomonas aeruginosa*	*Pseudomonas aeruginosa*	Negative	Negative	Negative	Negative
N4	*Staphylococcus aureus*	*Staphylococcus aureus*	Negative	Negative	Negative	Negative
N5	*Bacteroides fragilis*	Negative (normal flora)	Positive	Positive	Positive	Positive
N6	*Bacteroides fragilis*, *Escherichia coli*	Negative (normal flora)	Negative	Negative	Negative	Positive
N7	*Enterococcus faecalis*	Negative (normal flora)	Negative	Negative	Negative	Negative
N8	*Enterococcus faecalis*	Negative (normal flora)	Negative	Negative	Negative	Negative
N9	*Enterococcus faecalis*	Negative (normal flora)	Negative	Negative	Negative	Negative
N10	*Enterococcus faecium*	Negative (normal flora)	Negative	Negative	Negative	Negative
N11	*Escherichia coli*	Negative (normal flora)	Negative	Negative	Negative	Negative
N12	*Escherichia coli*	Negative (normal flora)	Negative	Negative	Negative	Negative
N13	*Escherichia coli*	Negative (normal flora)	Negative	Negative	Negative	Negative
N14	*Escherichia coli*	Negative (normal flora)	Negative	Negative	Negative	Negative
N15	*Escherichia coli*	Negative (normal flora)	Negative	Negative	Negative	Negative
N16	*Klebsiella pneumoniae*	Negative (normal flora)	Negative	Negative	Negative	Negative
N17	*Proteus mirabilis*	Negative (normal flora)	Positive	Negative	Negative	Negative
N18	*Proteus mirabilis*	Negative (normal flora)	Negative	Negative	Negative	Negative
N19	*Staphylococcus aureus*	Negative (normal flora)	Negative	Negative	Negative	Negative
N20	*Staphylococcus aureus*	Negative (normal flora)	Negative	Negative	Negative	Negative
N21	*Staphylococcus aureus*	Negative (normal flora)	Negative	Negative	Negative	Negative
N22	*Staphylococcus aureus*	Negative (normal flora)	Negative	Negative	Negative	Negative
N23	*Staphylococcus epidermidis*	Negative (normal flora)	Negative	Positive	Negative	Negative
N24	*Staphylococcus epidermidis*	Negative (normal flora)	Negative	Negative	Negative	Negative
N25	*Staphylococcus hominis*	Negative (normal flora)	Negative	Negative	Negative	Negative
N26	Negative	*Haemophilus influenzae*	Negative	Negative	Negative	Negative
N27	Negative	*Haemophilus influenzae*	Negative	Positive	Negative	Positive
N28	Negative	*Haemophilus influenzae*	Negative	Negative	Negative	Negative

Sample	Blood culture result	Respiratory tract culture result	UAT result			
			**BinaxNOW**	**ImmuView**	**STANDARD F**	**Sofia**
N29	Negative	*Haemophilus influenzae*	Negative	Negative	Negative	Negative
N30	Negative	*Haemophilus influenzae*	Negative	Negative	Negative	Negative
N31	Negative	*Haemophilus influenzae*	Positive	Positive	Positive	Positive
N32	Negative	*Klebsiella pneumoniae*	Positive	Positive	Positive	Positive
N33	Negative	*Moraxella catarrhalis*	Negative	Positive	Negative	Negative
N34	Negative	*Moraxella catarrhalis*	Negative	Negative	Negative	Negative
N35	Negative	*Moraxella catarrhalis*	Negative	Negative	Negative	Negative
N36	Negative	*Moraxella catarrhalis*	Negative	Negative	Negative	Negative
N37	Negative	*Moraxella catarrhalis*	Negative	Negative	Negative	Negative
N38	Negative	*Moraxella catarrhalis*	Positive	Positive	Positive	Positive
N39	Negative	*Pseudomonas aeruginosa*	Negative	Negative	Negative	Negative
N40	Negative	Negative (normal flora)	Negative	Negative	Negative	Negative
N41	Negative	Negative (normal flora)	Negative	Negative	Negative	Negative
N42	Negative	Negative (normal flora)	Negative	Negative	Negative	Negative
N43	Negative	Negative (normal flora)	Negative	Positive	Negative	Positive
N44	Negative	Negative (normal flora)	Negative	Negative	Negative	Negative
N45	Negative	Negative (normal flora)	Negative	Negative	Negative	Negative
N46	Negative	Negative (normal flora)	Negative	Negative	Negative	Negative
N47	Negative	Negative (normal flora)	Positive	Positive	Positive	Positive
N48	Negative	Negative (normal flora)	Negative	Negative	Negative	Negative
N49	Negative	Negative (normal flora)	Negative	Negative	Negative	Negative
N50	Negative	Negative (normal flora)	Negative	Negative	Negative	Negative
N51	Negative	Negative (normal flora)	Negative	Negative	Negative	Negative
N52	Negative	Negative (normal flora)	Negative	Negative	Negative	Negative
N53	Negative	Negative (normal flora)	Negative	Negative	Negative	Negative
N54	Negative	Negative (normal flora)	Negative	Negative	Negative	Negative
N55	Negative	Negative (normal flora)	Negative	Negative	Negative	Negative
N56	Negative	Negative (normal flora)	Negative	Negative	Positive	Invalid
N57	Negative	Negative (normal flora)	Negative	Negative	Negative	Negative
N58	Negative	Negative (normal flora)	Negative	Negative	Negative	Negative

All urine samples were collected from patients with simultaneously negative nasopharyngeal and/or lower respiratory tract cultures for *Streptococcus pneumoniae*.

Table S3. Results of *Streptococcus pneumoniae* UATs after re-freezing and 4 months of storage

			**UAT result**		
**Sample**	**Blood culture result**	**Respiratory tract culture result**	**BinaxNOW**	**ImmuView**	**STANDARD F**
P3	*Streptococcus pneumoniae*	*Streptococcus pneumoniae*, *Staphylococcus aureus*	Negative	Negative*	Negative
P14	*Streptococcus pneumoniae*	*Streptococcus pneumoniae*	Positive	Positive	Positive
P15	*Streptococcus pneumoniae*	*Streptococcus pneumoniae*	Negative	Negative	Negative
P16	*Streptococcus pneumoniae*	*Streptococcus pneumoniae*	Positive	Positive	Positive
P17	*Streptococcus pneumoniae*	*Moraxella cattarhalis*	Positive	Positive	Positive
P19	*Streptococcus pneumoniae*	*Staphylococcus aureus*	Positive	Negative	Positive
P20	*Streptococcus pneumoniae*	*Staphylococcus aureus*	Positive	Positive	Positive
P23	*Streptococcus pneumoniae*	Negative (normal flora)	Negative	Negative	Negative
P24	*Streptococcus pneumoniae*	Not determined	Negative	Negative	Negative
P25	*Streptococcus pneumoniae*	Not determined	Positive	Positive	Negative
P26	*Streptococcus pneumoniae*	Not determined	Positive	Positive	Positive
P27	*Streptococcus pneumoniae*	Not determined	Negative	Negative	Negative
P29	*Streptococcus pneumoniae*	Not determined	Positive	Positive	Positive
P30	*Streptococcus pneumoniae*	Not determined	Positive	Positive	Positive
P31	*Streptococcus pneumoniae*	Not determined	Positive	Positive	Positive
P32	*Streptococcus pneumoniae*	Not determined	Positive	Positive	Positive
P33	*Streptococcus pneumoniae*	Not determined	Positive	Positive	Positive
P34	*Streptococcus pneumoniae*	Not determined	Positive	Positive	Positive
P35	*Streptococcus pneumoniae*	Not determined	Positive*	Negative	Positive*
P49	Negative	*Streptococcus pneumoniae*	Positive	Positive	Positive
N3	*Pseudomonas aeruginosa*	*Pseudomonas aeruginosa*	Negative	Negative	Negative
N6	*Bacteroides fragilis*, *Escherichia coli*	Negative (normal flora)	Negative	Positive*	Negative
N14	*Escherichia coli*	Negative (normal flora)	Negative	Negative	Negative
N17	*Proteus mirabilis*	Negative (normal flora)	Negative*	Negative	Positive*
N18	*Proteus mirabilis*	Negative (normal flora)	Negative	Negative	Negative
N23	*Staphylococcus epidermidis*	Negative (normal flora)	Negative	Positive	Negative
N26	Negative	*Haemophilus influenzae*	Negative	Negative	Negative
N39	Negative	*Pseudomonas aeruginosa*	Negative	Negative	Negative

			UAT result		
**Sample**	**Blood culture result**	**Respiratory tract culture result**	**BinaxNOW**	**ImmuView**	**STANDARD F**
N46	Negative	Negative (normal flora)	Negative	Negative	Negative
N47	Negative	Negative (normal flora)	Positive	Positive	Positive
N48	Negative	Negative (normal flora)	Negative	Negative	Negative
N49	Negative	Negative (normal flora)	Negative	Negative	Negative
N50	Negative	Negative (normal flora)	Negative	Negative	Negative
N51	Negative	Negative (normal flora)	Negative	Negative	Negative
N52	Negative	Negative (normal flora)	Negative	Negative	Negative
N53	Negative	Negative (normal flora)	Negative	Negative	Negative
N56	Negative	Negative (normal flora)	Negative	Positive*	Positive

* changed test results after re-freezing and storage
